# Quantitative biomechanical assessment of locomotor capabilities of the stem archosaur *Euparkeria capensis*

**DOI:** 10.1098/rsos.221195

**Published:** 2023-01-25

**Authors:** Oliver E. Demuth, Ashleigh L. A. Wiseman, John R. Hutchinson

**Affiliations:** ^1^ Structure and Motion Laboratory, Department of Comparative Biomedical Sciences, The Royal Veterinary College, Hatfield, UK; ^2^ Department of Earth Sciences, University of Cambridge, Cambridge, UK; ^3^ McDonald Institute for Archaeological Research, University of Cambridge, Cambridge, UK

**Keywords:** locomotion, musculoskeletal modelling, Archosauria, bipedalism

## Abstract

Birds and crocodylians are the only remaining members of Archosauria (ruling reptiles) and they exhibit major differences in posture and gait, which are polar opposites in terms of locomotor strategies. Their broader lineages (Avemetatarsalia and Pseudosuchia) evolved a multitude of locomotor modes in the Triassic and Jurassic periods, including several occurrences of bipedalism. The exact timings and frequencies of bipedal origins within archosaurs, and thus their ancestral capabilities, are contentious. It is often suggested that archosaurs ancestrally exhibited some form of bipedalism. *Euparkeria capensis* is a central taxon for the investigation of locomotion in archosaurs due to its phylogenetic position and intermediate skeletal morphology, and is argued to be representative of facultative bipedalism in this group. However, no studies to date have biomechanically tested if bipedality was feasible in *Eupakeria*. Here, we use musculoskeletal models and static simulations in its hindlimb to test the influences of body posture and muscle parameter estimation methods on locomotor potential. Our analyses show that the resulting negative pitching moments around the centre of mass were prohibitive to sustainable bipedality. We conclude that it is unlikely that *Euparkeria* was facultatively bipedal, and was probably quadrupedal, rendering the inference of ancestral bipedal abilities in Archosauria unlikely.

## 1. Introduction

Shifts in locomotor modes and postures, from quadrupedal to bipedal, or vice versa, were pivotal in the evolution of tetrapods [1–6]. While these postural shifts are relatively rare, bipedalism has evolved independently in Squamata and Mammalia, and multiple times within Archosauria [1,5,7–10]. Birds and crocodylians, the last surviving lineages of Archosauria, are polar opposites in terms of posture and gait, the former being a habitual and erect biped and the latter a quadruped with a more sprawling-type gait [11–20]. However, throughout their evolutionary history, the two archosaurian clades Avemetatarsalia and Pseudosuchia explored a multitude of different locomotor modes [21–27], including multiple occurrences of bipedalism, such as their independent acquisition in pseudosuchians (i.e. poposaurids and shuvosaurids) and *Postosuchus*, and in Avemetatarsalia perhaps in lagerpetids and pterosauromorphs, such as *Scleromochlus* [9,10,28–31]. The abundant fossil record of archosaurs facilitates detailed studies of their morphological and locomotor evolution [2,21,31–33]. However, the exact timing and frequency of the emergence of a more upright gait and bipedalism remain contentious [2,10,21,25–27,31,34,35].

**Figure 1.**
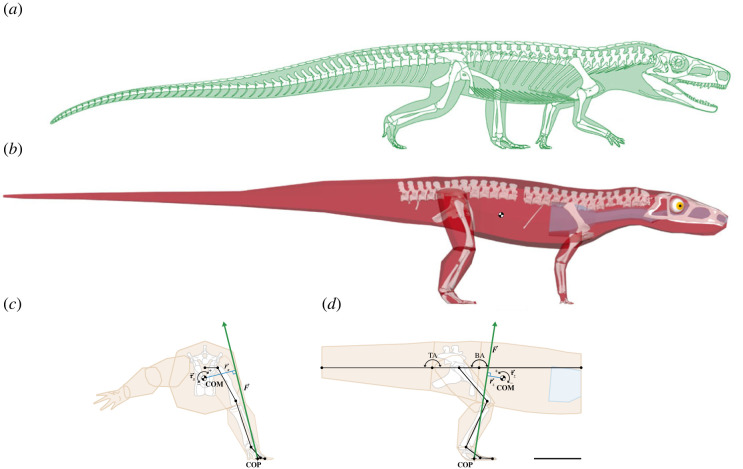
*Euparkeria* model. (*a*) Skeletal reconstruction of *Euparkeria capensis*, modified from Cuff *et al*. [27]. (*b*) Articulated digital skeleton of *Euparkeria* and hull and cavity models used for centre of mass (COM) (crossed circle) and inertia calculations. Modelled body posture and ground reaction force (GRF) vector in caudal view (*c*) and right lateral view (*d*). The body segments are shown in orange and the body cavities (lung, trachea, cranial sinus/pharynx) in blue; dots denote modelled joint centres. Note that the COM is cranial to both the hip joint and the GRF vector. ***r_x_*** and ***r_z_***, respectively, represent the roll and pitch moment arms of the GRF vector ***F*** about the COM, producing the COM roll and pitch moments ***τ_x_*** and ***τ_z_***. COM, centre of mass; COP, centre of pressure; BA, body angle; TA, tail angle. Angle deviations in the body and tail were measured from horizontal. All drawings to scale, scale bar = 5 cm.

The early archosauriform *Euparkeria capensis* from the Middle Triassic of South Africa [33,36] is a key taxon for disentangling the early locomotor evolution of archosaurs due to its plesiomorphic skeletal morphology, i.e. small-bodied, gracile, terrestrial and cursorial (see [37]; figure 1*a*), with a presumed posture that was intermediate between that of earlier, sprawling taxa and the fully erect stance seen in later taxa [25–27,37]. Together, these traits resemble the expected ancestral body plan for Archosauria [36–38]. This ancestral form and function are further supported by its stable phylogenetic position as sister taxon to Archosauria in both Bayesian and parsimony-based analyses [37,39]. The exact locomotor mode of *Euparkeria* has been a subject of debate. It has been classified as a facultative biped [9,24,33,40], mainly based on limb proportions. Contrastingly, other studies have instead classified *Euparkeria* as a habitual quadruped [10,30] with a relatively sprawling posture based on skeletal forelimb [41], hindlimb and ankle morphology [33,42], or alternatively with a more erect limb posture due to the similarity of the hindlimb bones and joints with those of crocodylians [21,22,43] or quantitative and functional analyses of the hindlimbs [26]. While joint mobility of its hindlimbs [26] or its muscle leverage in a comparative context [27] has been investigated, the locomotor performance of *Euparkeria* remains to be quantitatively tested.

Herein, we aim to overcome previous qualitative locomotory reconstructions, based on limb bone morphologies and their comparisons with potentially (but not necessarily) analogous taxa and/or somewhat ambiguous assessments solely based on limb proportions, using quantitative and biomechanical principles. Musculoskeletal models can provide valuable insights into musculoskeletal function, which cannot otherwise be directly measured in physical experiments [20,44], and have become an increasingly popular tool in palaeobiology to estimate locomotor function and biomechanics [27,44–46]. A holistic approach, combining musculoskeletal modelling and other biomechanical tools, such as mechanical modelling of pitch moments around the centre of mass (COM) (e.g. [47,48]), enables the testing of bipedal capabilities in *Euparkeria*.

Forward acceleration generates a pitch moment around the COM and lifts the forelimbs off the ground, thus inducing bipedal postures, and that acceleration ends as soon as the velocity becomes constant and, consequently, the pitch moment dissipates [47,49]. Acceleration-induced bipedalism is, therefore, inherently unsustainable. By contrast, recent theoretical and experimental studies have shown that temporal asymmetry in the ground reaction force (GRF), in which the vertical component is skewed so that higher forces are exerted early in the stance phase, play a crucial role in achieving steady bipedal locomotion in birds and lizards [8,15,48,49], because their COM is situated cranial to the hips. An early skewed GRF profile increases the total amount of force that is applied along a resultant vector cranial to the COM during a stride, resulting in a larger nose-up pitch moment around the hips [8,48,49]. Additionally, angular acceleration of the trunk and tail about the hips are important factors in destabilizing quadrupedalism and facilitating bipedal locomotion instead [1,48]. Other forces such as aerodynamic lift play only a minor, effectively negligible role [49].

Here, we used musculoskeletal modelling and static simulations to investigate the bipedal capabilities of *E. capensis* and to test (i) if this species could have sustained a peak GRF expected during bipedal locomotion, and (ii) if the pitching moment about the COM facilitated sustainable bipedal locomotion. We then revisit the potential stance (habitually quadrupedal, facultatively or habitually bipedal) of ancestral Archosauria in light of our conclusions.

## 2. Material and methods

### 2.1. Model creation

The digital skeleton of *E. capensis* was assembled as composite of four µCT-scanned specimens (table 1) which were scaled to the holotype SAM PK 5867, the most complete specimen [26,33]. The bones were segmented in Avizo 9.7 Lite (Thermo Fisher Scientific Inc., Waltham, MA, USA; https://www.thermofisher.com/uk/en/home/electron-microscopy/products/software-em-3d-vis/avizo-software.html) and Materialise Mimics 22.0 (Materialise NV, Leuven, Belgium; https://www.materialise.com/en/healthcare/mimics-innovation-suite/mimics) and rearticulated in a standard reference pose [16,50,51] in Autodesk Maya 2019 (Autodesk Inc., San Rafael, CA, USA; https://www.autodesk.com/products/maya/overview) following anatomical and joint coordinate system creation using geometric primitive shape-fitting [26,50,51]. The majority of the tail is not preserved in any of the µCT-scanned specimens of *Euparkeria*; it was, therefore, reconstructed based on photographs of several additional specimens (table 1; electronic supplementary material, figure S1). The model was posed in an osteologically feasible posture [26] for body segment modelling and COM estimation [50,52,53] and volumetric muscle creation [54,55] (figure 1*b*). We used a previously published three-dimensional musculoskeletal reconstruction of *Euparkeria* [27,55], which is briefly described here. Muscle origins and insertions were identified based on muscle scarring the extant phylogenetic bracket (EPB) [56,57] in combination with ancestral state reconstruction for ambiguous muscles following Bishop *et al*. [50] (see electronic supplementary material, figure S2 for muscle map). *Euparkeria* was, therefore, rescored based on new observations in the revised matrix [50] for osteological correlates of hindlimb musculature initially outlined by Hutchinson [58] and ambiguous muscle states reconstructed via maximum-parsimony analysis in Mesquite 3.40 [59] (http://www.mesquiteproject.org). Volumetric muscles were subsequently created in Autodesk Maya 2019, constrained by tomographic sections of alligator hindlimbs [55] due to their similarity in limb morphology and proportions [33] and evolutionarily conserved myology [55], and their lines of action estimated following previously established protocols [20,55,60].

**Table 1.** Specimen list. See Demuth *et al*. [26] for µCT scan parameters. Multiple specimens of *Euparkeria* are preserved on slab SAM PK K8050 and two of them have the proximal caudal vertebrae preserved in articulation (see electronic supplementary material, figure S1). Institutional abbreviations: SAM, Iziko South African Museum, Cape Town, South Africa; IFGT, Institute for Geosciences, Eberhard-Karls-Universität Tübingen, Tübingen, Germany (formerly Geologisch-Paläontologisches Institut Tübingen: GPIT); UMZC, University Museum of Zoology Cambridge, Cambridge, UK.

µCT-scanned specimens		specimen photographs	
specimen	elements	specimen	elements
SAM PK 5867	full body	SAM PK K8050 (1)	caudal vertebrae
SAM PK 6047A	pelvic girdle and forelimbs	SAM PK K8050 (2)	caudal vertebrae
SAM PK K8309	hindlimbs	SAM PK 13666	caudal vertebrae
UMZC T.692	fore and hindlimbs	GPIT 1686.1	caudal vertebrae

### 2.2. Musculoskeletal simulations

GRFs and body segment orientations were directly informed by the kinetics and kinematics of lizards [49,61–63]. This is unlike previous studies, for which the GRF was directed vertically from exactly underneath the COM during mid-stance (e.g. [45,46,50]); however, this was not feasible because of the less erect hindlimb posture of *Euparkeria* (see [26]). Additionally, considering the asymmetrical GRFs in extant bipedally running lizards that peak during early stance phase [48], here it was assumed that the GRF profile for a bipedal *Euparkeria* would also have peaked during early stance. The hindlimb poses were configured accordingly and the GRF was thus applied to the centroid of the contact area (COP; figure 1*c*,*d*) of the right foot during early stance for the static simulations (no dynamic movement was modelled, see below). The assumed limb orientation was informed by data for the early stance phase in bipedally running lizards, using running kinematics of *Dipsosaurus dorsalis* and *Callisaurus draconoides* [61] (see electronic supplementary material, information S4 for joint angles). The GRF vector was adopted from empirical data collected by Sheffield *et al*. [62] and thus aimed 14° medially and 8° cranially (figure 1*c*,*d*). To test if *Euparkeria* could sustain GRFs expected during bipedal locomotion and how the body postures influenced the resulting muscle activations and pitch moments around the COM, several simulations and musculoskeletal models were set up. To cover the full breadth of possible bipedal body postures, the trunk and tail angles were each rotated by 0°, 25°, 50° and 75° (from horizontal to almost vertical), resulting in 16 simulations with varying combinations of trunk and tail angles (see [49]).

The models each had 16 active degrees of freedom, three rotational (pitch, yaw and roll) and three translational d.f. in the pelvis, three rotational d.f. (flexion/extension, abduction/adduction and long-axis rotation) in the hip and one d.f. (flexion/extension) each in the knee, ankle and metatarsophalangeal joints, and two d.f. (pitch and yaw) in both the proximal and mid-tail joints. Additionally, there were a total of three fixed (locked) d.f. per model: one d.f. (pitch) each in the trunk, neck and head joints.

To test the sensitivity of the architectural muscle parameters on the simulation results, four OpenSim [64] (https://simtk.org/projects/opensim) musculoskeletal models were created using different estimation methods for muscle parameters (maximal isometric force *F*_max_ and optimal fibre length *l_o_*): Model 1, alpha shape centroid; Model 2, convex hull centroid; and Model 3, arithmetic centroid; all following Bishop *et al*. [50] using previously published saurian muscle architectural data [55,65–67] representing the EPB [56,57]. Model 4 was generated from three-dimensional volumetric muscle models following Demuth *et al*. [55] (see electronic supplementary material, informations S1 and S2 for all muscle parameters). The muscle parameters were normalized and scaled by body mass [67–69], rather than segment or muscle tendon unit length [50], for data comparability. For the 16 static inverse simulations in OpenSim 3.3 [64], the magnitude of the GRF was systematically increased for each pose until the static optimization algorithm could no longer find a solution to achieve static equilibrium for any combination of muscle activations (*a_m_*), while minimizing the sum of squared activations (see [50]),

2.1 $$\min\left(\overset{N}{\underset{m=1}{\sum }}a_{m}^{2}+\overset{Q}{\underset{q=1}{\sum }}a_{q}^{2}\right),$$

2.2 $$\mathrm{subject}\;\mathrm{to}\;\overset{n}{\underset{i=1}{\sum }}F_{i}\cdot r_{i,k}+M_{r,k}=\;M_{k}$$

2.3 $$\mathrm{and}\;\;F_{i}=a_{i}\cdot F_{\max,i},$$

for each degree of freedom *k* (=16). There were *N* = 36 muscles (electronic supplementary material, figure S2 and information S2) and *Q* = 5 reserve actuators in each model (one at the metatarsophalangeal joint (flexion/extension) and two each in the proximal and mid-tail joints (roll and yaw)). The maximal torques of these reserve actuators are inconsequential as long as they are sufficiently large enough to never be fully activated [45,50]. Muscle force *F_i_* was modelled as the product of activation and *F*_max_, while ignoring any intrinsic force–length–velocity relationships [50] (electronic supplementary material, information S2). The moment each muscle produced about a given degree of freedom was the product of its force *F_i_* and its moment arm *r_i,k_*. In total, 64 static simulations were computed, each testing a combination of the four different joint poses for both the tail and body and the different muscle parameter estimation methods in the four models.

The resulting muscle activations were interpolated at 1° intervals between the four different body postures per model, using cubic spline interpolation in Matlab 2020a (The MathWorks, Inc., Natick, MA, USA; https://www.mathworks.com/products/matlab.html) to produce full coverage of the simulation space. Tail orientation had no influence on muscle activation and was, therefore, omitted.

### 2.3. COM moment calculations

The pitching moments around the COM were determined using the geometric definition of the moment arm as being the shortest perpendicular distance between a force's line of action and a centre of rotation [70], i.e. the COM position. Each moment was calculated as the shortest distance between two skew lines: the GRF vector from the COP in the foot and the unit vectors from the COM position representing its principal axes (figure 1*c*,*d*), and scaled by the two-dimensional projection of that distance onto each axis's plane of rotation [71], i.e. using its vector components (electronic supplementary material, information S3). The COM moments ***τ*** were then calculated as the cross product of the moment arm ***r*** and the GRF ***F*** with a magnitude of 2 body weights (BWs),

2.4 τ=r×F.

The moments around the COM were interpolated at 1° intervals between the 16 different simulations using cubic spline interpolation in Matlab 2020a to cover the full range of tested postures.

The body segment orientations of six extant lizard species during bipedal locomotion were qualitatively compared with *Euparkeria* to relate their postural diversity to the pitching moments computed for *Euparkeria*. Data for lizards were gathered from Olberding *et al*. [63] for *Aspidoscelis sexlineata*, Irschick & Jayne [61] for *Callisaurus draconoides*, *Cnemidophorus tigris*, *Dipsosaurus dorsalis* and *Uma scoparia*, and van Wassenbergh & Aerts [49] for *Ctenophorus cristatus*.

### 2.4. Ancestral state reconstruction

The evolution of bipedalism within Archosauria was further investigated based upon two hypotheses regarding their interrelationship: the first phylogenetic tree, termed ‘Nesbitt tree’ followed work by Nesbitt [72], Nesbitt *et al*. [73], von Baczko *et al*. [74], Ezcurra *et al*. [75] and Foffa *et al*. [31], and the second, alternative tree, termed ‘Ezcurra tree’ was based on recent work by Ezcurra [76], Garcia *et al*. [77] and Müller & Garcia [78]. The phylogenetic trees were computed in the open-source software R [79] using the function ‘DatePhylo’ in the ‘strap’ package [80] and ancestral states were computed using different maximum-likelihood models, following Grinham *et al.* [9] in the R packages ‘ape’ [81] and ‘phytools’ [82]. The character states of the individual taxa were defined following [10,50]. The tips for the ambiguous taxa, i.e. *Hesperosuchus*, Pterosauria, Lagerpetidae, *Scleromochlus* and Silesauridae, were additionally estimated using the different models with their priors set to 0.5 for two character states (quadrupedal, bipedal) and 0.333 for the models with three character states (quadrupedal, facultatively bipedal, bipedal). For the latter, the transition matrices were manually set to ensure that the character states were ordered (from quadrupedal to facultatively bipedal to bipedal and vice versa). The most likely model was then chosen based on the Akaike information criterion (AIC) [83]. See electronic supplementary material, data for the R code and data.

## 3. Results

### 3.1. Pitch moments

**Figure 2.**
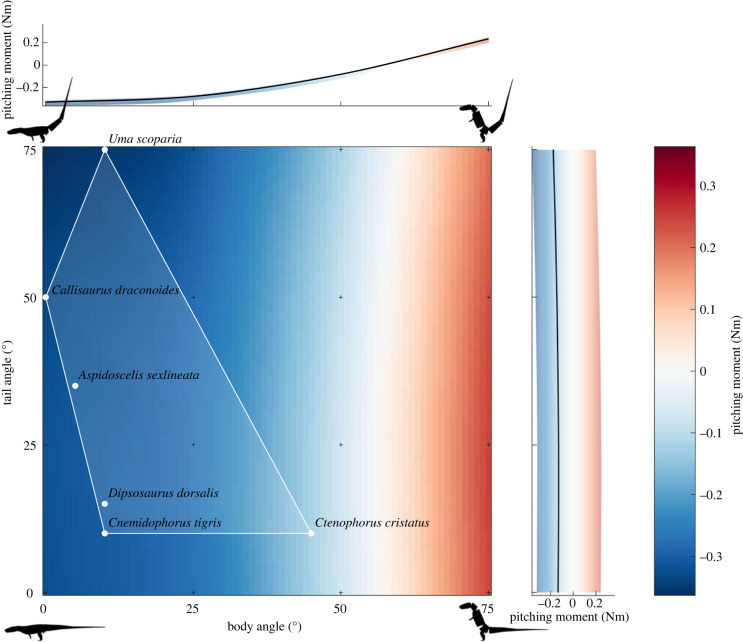
Pitching moment around the COM of *Euparkeria* during early stance and asymmetrical GRF. The black silhouettes qualitatively describe the body orientations of *Euparkeria* at the extreme points of the simulations. Body and tail postures of six extant lizards during bipedal locomotion are illustrated as circles and the posture space they occupy is indicated by the white transparent convex hull. Positive pitching moments (nose-up) are red, while negative pitching moments (nose-down) are blue. Note that bipedal poses similar to extant lizards resulted in a negative pitching moment around the COM for *Euparkeria*. Crosses denote simulation results; the values in between were interpolated at 1° intervals. The top and side plots display the range and average of pitching moments in relation to the body and tail angles, respectively.

Most of *Euparkeria*'s mass was concentrated in front of the hips, and the effects of changes in the static, plausible tail angles on the pitch moment mostly were negligible (s.d. ± 0.014 Nm; figure 2), whereas increase of the body angle shifted the COM towards the hips and substantially influenced the pitch moments (s.d. ± 0.175 Nm; figure 2). The magnitude of the negative pitching moment (nose-down) decreased with increasing body angle, until it switched sign, approximately at a body angle of 60°, and then subsequently increased again. The calculated pitch moments ranged from −0.363 Nm in the posture with a 0° body angle and 75° tail angle, forcing the COM cranially (nose-down), to 0.246 Nm where the body and tail angles were 75° and 25°, respectively, forcing the COM caudally and dorsally (nose-up; figure 2). The six extant bipedal lizards all occupied postures with a large negative pitch moment for *Euparkeria* (figure 2). Only the posture of *Ctenophorus cristatus*, which has a more inclined body, approached a more neutral pitch moment; albeit still negative.

Sensitivity testing the modelling assumptions for the COM calculations, modifying the body segment shapes by ±20% [52,53,84] and pushing the segment masses and their COM to implausible extremes [84], did not substantially influence our results (electronic supplementary material, figure S3 and information S3). The pitch moment around the COM stayed positive only for the implausible maximally caudal hull throughout the different body and tail orientations (electronic supplementary material, figure S3 and information S3). The overall mean across all hulls for the sensitivity analysis was consistent with the optimal hull, with a mean difference of the pitching moments of only 4.85% (electronic supplementary material, information S3), indicating that the optimal hull (as presented in figure 2) best represented the body proportions and mass properties of *Euparkeria*.

### 3.2. Muscle activation

**Figure 3.**
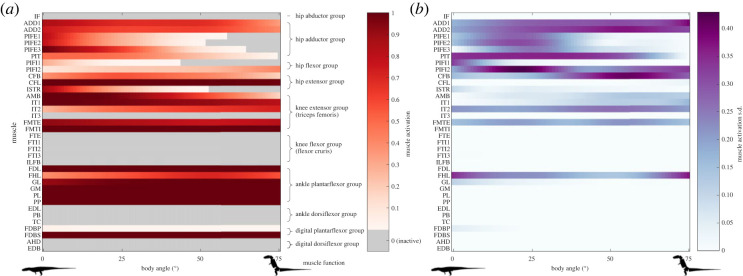
Mean activation (*a*) and its corresponding standard deviation (*b*) of the *Euparkeria* muscles across all models and simulations at their maximally sustainable GRF. Tail angle had no influence on muscle activation. Mean muscle activation was calculated at 0°, 25°, 50° and 75°; the values in between were interpolated at 1° intervals. The distal hindlimb muscle homologies follow Hattori & Tsuihiji [85]. See electronic supplementary material, figure S4 for the muscle activation of the individual models. IF, M. iliofemoralis; ADD 1–2, M. adductor femoris 1 + 2; PIFE1–3, M. puboischiofemoralis externus 1–3; PIT, M. puboischiotibialis; PIFI1–2, M. puboischiofemoralis internus 1–2; CFB, M. caudofemoralis brevis; CFL, M. caudofemoralis longus; ISTR, M. ischitrochantericus; AMB, M. ambiens; IT1–3, M. iliotibialis 1–3; FMTE, M. femorotibialis externus; FMTI, M. femorotibialis internus; FTE, M. flexor tibialis externus, FTI1–3, M. flexor tibialis internus 1–3; ILFB, M. iliofibularis; FDL, M. flexor digitorum longus; FHL, M. flexor hallucis longus; GL, M. gastrocnemius lateralis; GM, M. gastrocnemius medialis; PL, M. peroneus (fibularis) longus; PP, M. pronator profundus; EDL, M. extensor digitorum longus; PB, M. peroneus (fibularis) brevis; TC, M. tibialis cranialis; FDBP, M. flexor digitorum brevis profundus; FDBS, M. flexor digitorum brevis superficialis; AHD, M. adductor hallucis dorsalis; EDB, M. extensor digitorum brevis.

The patterns in hindlimb muscle activations at peak GRF were roughly similar between the different simulations (figure 3; table 2; electronic supplementary material, figure S4). These muscle activation patterns generally fall within predicted ranges (see [50]). Muscles that counteract gravitational forces, e.g. the ankle plantarflexors, the knee extensors, the hip extensors and to some degree the hip adductors, were highly activated, while the antagonistic flexor muscles remained mostly inactive. The variation and discrepancy between the different simulations were highest among the pelvic muscles (figure 3*b*), mainly caused by Model 4, which had very high muscle activations in the adductor muscle group at low body angles, and additionally high activation in the hip flexor muscles, which were mostly inactive in the other models. Those activations were far less pronounced in the other simulations (table 2; electronic supplementary material, figure S4). In addition to the ankle plantarflexor muscles, the knee extensor muscles were also maximally recruited in Model 3, even surpassing the muscle activation of the ankle plantarflexors. Overall, the highest activations were observed in the ankle plantarflexor group, which was the ‘weak link’ in the simulations (figure 3*a*). A further increase of the GRF by 0.1 BW resulted in failure of those muscles and the collapse of the ankle joint in all simulations (see electronic supplementary material, table S1 for hind limb joint moments).

**Table 2.** Mean muscle activations per muscle group at maximal sustainable GRF. IT3 was excluded from knee extensors, because it acted mainly as knee abductor and knee flexor in the *Euparkeria* model and therefore was inactive during the simulations. Note that the ‘weak link’ was the ankle joint in most simulations; additionally, in Model 3 the knee extensors reached their limit and in Model 4 the hip adductors. abd., abductors; add., adductors; flex., flexors; ext., extensors; plan., plantar flexors; dorsi., dorsiflexors; dig., digital.

muscle groups	Model 1 (alpha shape centroid)				Model 2 (convex hull centroid)				Model 3 (arithmethic centroid)				Model 4 (three-dimensional model)			
body angle (°)	0	25	50	75	0	25	50	75	0	25	50	75	0	25	50	75
hip abd.	0.000	0.000	0.000	0.000	0.000	0.000	0.000	0.000	0.000	0.000	0.000	0.007	0.000	0.000	0.000	0.000
hip add.	0.624	0.302	0.144	0.052	0.762	0.334	0.193	0.081	0.765	0.415	0.282	0.076	1.000	0.901	0.494	0.287
hip flex.	0.090	0.020	0.021	0.060	0.069	0.000	0.046	0.090	0.041	0.000	0.268	0.413	0.724	0.431	0.131	0.273
hip ext.	0.718	0.559	0.395	0.331	0.801	0.633	0.460	0.362	0.790	0.679	0.667	0.532	0.727	0.574	0.400	0.333
knee ext.	0.773	0.755	0.695	0.638	0.891	0.870	0.801	0.743	0.885	0.954	0.957	0.853	0.800	0.859	0.692	0.588
knee flex.	0.000	0.000	0.000	0.000	0.000	0.000	0.000	0.000	0.000	0.000	0.000	0.000	0.000	0.000	0.000	0.000
ankle plan.	0.954	0.954	0.954	0.954	0.938	0.938	0.938	0.938	0.834	0.863	0.927	0.840	0.792	0.941	0.980	0.980
ankle dorsi.	0.000	0.000	0.000	0.000	0.000	0.000	0.000	0.000	0.000	0.000	0.000	0.000	0.000	0.000	0.000	0.000
dig. plan.	0.502	0.503	0.502	0.502	0.502	0.503	0.502	0.502	0.502	0.502	0.503	0.503	0.705	0.503	0.502	0.502
dig. dorsi.	0.000	0.000	0.000	0.000	0.000	0.000	0.000	0.000	0.000	0.000	0.000	0.000	0.000	0.000	0.000	0.000
max GRF [BW]	2.9	2.9	2.9	2.9	3.0	3.0	3.0	3.0	2.9	3.0	3.1	3.0	6.7	7.3	7.4	7.4

Changes in body angle mainly influenced the hip adductor and hip extensor muscle group activations. Alongside an increased body angle, the COM was located more caudally towards the pelvis, reducing its moment arm about the hip joint and thus becoming less demanding on those two muscle groups. Interestingly, an increase in the body angle shifted the muscle activation in the hip flexor group from PIFI1 to PIFI2 in all simulations; however, their activations remained relatively low overall (figure 3; electronic supplementary material, figure S4).

Due to the larger estimated muscle masses based on the three-dimensional models (electronic supplementary material, information S2) and the resultant associated *F*_max_ in Model 4, the sustained GRF was between 130% and 145% greater than in the other three models (table 2), for which the muscle parameters were solely estimated using scaled architectural muscle data from extant Sauria.

### 3.3. Evolution of bipedalism within Archosauria

**Figure 4.**
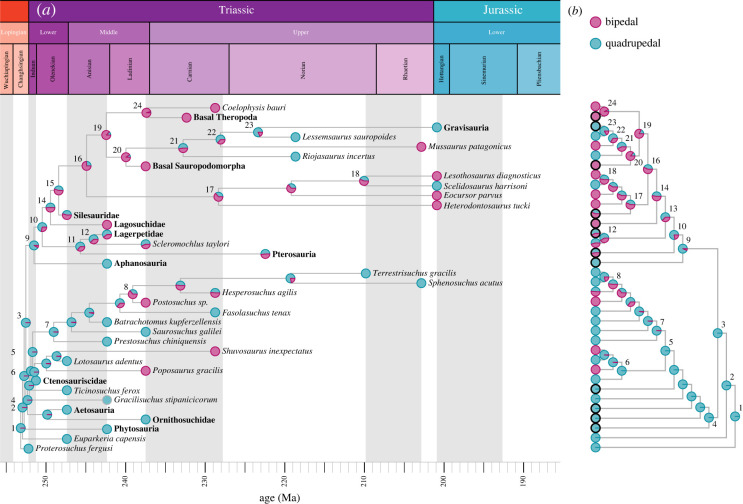
Evolution of bipedalism within Archosauria. Ancestral state reconstruction for the time-calibrated ‘Nesbitt tree’ (*a*) and ‘Ezcurra tree’ (*b*); see methods for sources of tree topologies; see electronic supplementary material, figures S5–S8 for individual time-calibrated trees. Red circles indicate bipedalism and blue circles indicate quadrupedalism; black outlined circles in (*b*) represent the bold clades in (*a*). Clades: 1, Archosauriformes; 2, Eucrocopoda; 3, Archosauria; 4, Pseudosuchia; 5, Paracrocodylomorpha; 6, Poposauroidea; 7, Loricata; 8, Crocodylomorpha; 9, Avemetatarsalia; 10, Ornithodira; 11, Pterosauromorpha; 12, Lagerpetidae; 13, Dinosauromorpha; 14, Dinosauriformes; 15, Dracohors; 16, Dinosauria; 17, Ornithischia; 18, Neornithischia; 19, Saurischia; 20, Sauropodomorpha; 21, Massopoda; 22, Sauropodiformes; 23; Sauropoda; 24, Theropoda.

The ancestral state reconstructions for both tree topologies were very similar (figure 4) and the results were thus relatively robust to differing hypotheses about their interrelationships or uncertainties in the tree topologies. Two character states with an ‘equal rates’ model were considered to be the most likely based on AIC values (49.69 for the ‘Nesbitt tree’ and 49.71 for the ‘Ezcurra tree’). For three character states an ‘unequal rates’ model with all rates different would have been the most likely (AIC 63.91 for the ‘Nesbitt tree’ and 63.54 for the ‘Ezcurra tree’); however, the models with three character states were all less likely than the models with only two character states (lower log-likelihood and larger AIC values; see electronic supplementary material, table S2).

Overall, Archosauria was consistently reconstructed as quadrupedal, and Pseudosuchia might have evolved bipedalism up to three times (perhaps twice in poposaurids; once in *Postosuchus*), all within the Ladinian–Carnian stages (Middle–Late Triassic). The estimation of Crocodylomorpha as ancestrally bipedal, however, might be an artefact of the short branch length leading to *Postosuchus* sp. In Avemetatarsalia, the emergence of bipedalism is somewhat ambiguous. It arose in the Olenekian, but there were some differences in the maximum likelihoods for its exact occurrence depending on the tree. Bipedalism arose once at the base of Dinosauriformes in the ‘Ezcurra tree’. Contrastingly, however, in the ‘Nesbitt tree’ it potentially emerged twice; firstly, in Dracohors, on the line leading to dinosaurs, and secondly, in parallel, in Lagosuchidae. Lagerpetids and pterosaurs consistently were estimated as quadrupedal, while the ambiguous Silesauridae was consistently estimated as ancestrally bipedal.

## 4. Discussion and conclusion

The hindlimb of *Euparkeria* was able to sustain the GRF expected during bipedal locomotion (greater than 2 BW) in all simulations, however, due to the large negative pitch moments around the COM at maximum GRF for most simulations, and especially in postures adopted by extant bipedal lizards (figure 2), it appears unlikely that *Euparkeria* habitually adopted bipedalism. To sustain bipedal locomotion, the angular momentum about the COM that is generated during each stride must be zero [15,47].

Temporal asymmetry in the GRF in birds and lizards, with higher forces exerted early in stance phase, enables steady bipedal locomotion, due to a larger nose-up pitch moment, resulting from a larger amount of force that is applied along the resultant vector cranial to the COM during a stride [8,15,47,48], and their COM is situated cranial to the hips. Under the assumption of such an early skewed GRF profile, resulting in a larger nose-up pitch moment [8,48,49], the pitch moment at peak GRF for *Euparkeria* should have been positive or close to zero if it were bipedal. However, this was only possible at extreme body angles of greater than 60° in our simulations, unknown in any bipedal saurian. Of the six extant bipedal lizard taxa for which body postures have been reported, *Euparkeria* qualitatively matches the body proportions of *Callisaurus draconoides*, which has the most negative pitch moments (figure 2), most closely. *Euparkeria* is, however, significantly larger than any of the bipedal lizard taxa, and, unfortunately but also intriguingly, no modern analogue exists for *Euparkeria*.

We did not model any dynamic movement, such as forward acceleration [7,47] or rotational inertia and angular momentum of the body and tail [1,47,48,86], which help counteract the nose-down pitch moment caused by gravity. However, such dynamic assumptions may not change our conclusions, which were based solely upon static simulations. In particular, an acceleration causing a positive pitching moment that lifts the trunk and the forelimbs off the ground only occurs when speed is increasing. As soon as the animal reaches its maximal speed, the net propulsive impulse and the resulting pitching moment become zero [47] and gravity takes over—pushing the nose back down (gravity-induced negative pitch; figure 2). Purely acceleration-driven bipedal locomotion is, therefore, inherently unsustainable and cannot explain bipedal locomotion in extant lizards [7,49,63]; hence acceleration was disregarded in this study. Dynamic simulations which incorporate angular momentum, such as Bishop *et al*. [87], may provide an avenue for future investigation to decipher the evolution of bipedal locomotor capabilities within archosaurs. Regardless, our findings are in line with recent quantitative studies on femoral shape [10], femoral microanatomy [88] and body COM position [30], suggesting that *Euparkeria* was a habitual quadruped. We further support this conclusion from the point of view of musculoskeletal biomechanics.

Our results from the ancestral state reconstruction indicate that bipedalism evolved once, maybe twice, somewhere within Ornithodira; but at least twice in pseudosuchians (poposaurids, *Postosuchus*). While secondary quadrupedalism was common in Ornithodira; i.e. several groups of ornithischians [5] as well as in sauropods independently evolved quadrupedalism; Pseudosuchia may or may not have ever reverted back to quadrupedalism from a bipedal form as our results are currently ambiguous in that regard. Further quantitative testing of bipedalism in some of the ambiguous taxa, i.e. *Hesperosuchus*, *Scleromochlus* and/or silesaurids, is necessary to disentangle the evolution of bipedalism within Archosauria.

*Euparkeria* and other early archosauriforms lacked morphological and functional specializations related to bipedal movement, such as having a fully erect hindlimb and/or parasagittal gait [2,25,26]. Their pes was also more specialized for quadrupedal locomotion [26,89], which is unlike the elongated and mediolaterally compressed tarsus and pes seen in bipedal taxa [29,90]. Additionally, the femora of archosauromorphs, including both early avemetatarsalians and pseudosuchians, further suggest that this group had a habitually quadrupedal posture prior to and initially after the split into bird- and crocodile-line archosaurs [10]. Our functional and biomechanical analysis of the locomotor mode of *Euparkeria* goes beyond these primarily morphological studies. It demonstrates, and explains via Newtonian mechanics, that bipedalism could not be achieved with the body plan of early archosaurs. More specifically, our analysis has allowed for the testing of the potential locomotor behaviour of an extinct stem archosaur, pointing towards an answer to the controversy over how *Euparkeria* moved. Morphological changes in the pelvis and hindlimb of later pseudosuchians and ornithodirans facilitated a parasagittal gait, bipedalism and digitigrade foot orientation to independently evolve at some points [10,26,27,29]. Ancestral archosaurs, therefore, did not exhibit bipedalism (figure 4). Ornithodira and Pseudosuchia inherited a body plan from Archosauria that subsequently was modified in the aftermath of the Permo-Triassic mass extinction, enabling novel locomotor modes such as bipedalism to evolve independently.

## Data Availability

The datasets generated and/or analysed during the current study are included in this published article (and its electronic supplementary material, information files) [91]. The OpenSim models and their corresponding simulation files, the R code and its files for the ancestral state reconstruction, and the *Euparkeria* pelvis, hindlimb and muscle models are available from the following figshare repository: https://figshare.com/s/73c07f4c9b930c25a3f9.
